# Spinopelvic Adaptations in Standing and Sitting Positions in Patients With Adult Spinal Deformity

**DOI:** 10.7759/cureus.28113

**Published:** 2022-08-17

**Authors:** Rami El Rachkidi, Abir Massaad, Eddy Saad, Georges Kawkabani, Karl Semaan, Julien Abi Nahed, Ismat Ghanem, Virginie Lafage, Wafa Skalli, Ayman Assi

**Affiliations:** 1 Department of Orthopedics and Traumatology, Hôtel-Dieu de France Hospital, Beirut, LBN; 2 Laboratory of Biomechanics and Medical Imaging, University of Saint Joseph, Beirut, LBN; 3 Technology Innovation Unit, Hamad Medical Corporation, Doha, QAT; 4 Department of Orthopaedics, Lenox Hill Hospital, New York, USA; 5 Institut de Biomécanique Humaine Georges Charpak, Arts et Métiers, Paris, FRA

**Keywords:** adult scoliosis, gait analysis, quality-of-life, spine flexibility, sitting, standing, adult spinal deformity

## Abstract

Purpose

To describe spinopelvic adaptations in the standing and sitting positions in patients with adult spinal deformity (ASD).

Methods

Ninety-five patients with ASD and 32 controls completed health-related quality of life (HRQOL) questionnaires: short form 36 (SF36), Oswestry Disability Index (ODI), and visual analog scale (VAS) for pain. They underwent biplanar radiography in both standing and sitting positions. Patients with ASD were divided into ASD-front (frontal deformity Cobb > 20°, n = 24), ASD-sag (sagittal vertical axis (SVA) > 50 mm, pelvic tilt (PT) > 25°, or pelvic incidence (PI)-lumbar lordosis (LL) > 10°, n = 40), and ASD-hyper thoracic kyphosis (TK >60°, n = 31) groups. Flexibility was defined as the difference (Δ) in radiographic parameters between the standing and sitting positions. The radiographic parameters were compared between the groups. Correlations between HRQOL scores were evaluated.

Results

All participants increased their SVA from standing to sitting (ΔSVA<0), except for patients with ASD-sag, who tended to decrease their SVA (78-62 mm) and maximize their pelvic retroversion (27-40° vs 10-34° in controls, p<0.001). They also showed reduced thoracic and lumbar ﬂexibility (ΔLL = 3.4 vs 37.1°; ΔTK = −1.7 vs 9.4° in controls, p<0.001). ASD-hyperTK showed a decreased PT while sitting (28.9 vs 34.4° in controls, p<0.001); they tended to decrease their LL and TK but could not reach values for controls (ΔLL = 22.8 vs 37.1° and ΔTK = 5.2 vs 9.4°, p<0.001). The ASD-front had normal standing and sitting postures. ΔSVA and ΔLL were negatively correlated with the physical component scale (PCS of SF36) and ODI (r = −0.39 and r = −0.46, respectively).

Conclusion

Patients with ASD present with different spinopelvic postures and adaptations from standing to sitting positions, with those having sagittal malalignment most affected. In addition, changes in standing and sitting postures were related to HRQOL outcomes. Therefore, surgeons should consider patient sitting adaptations in surgical planning and spinal fusion. Future studies on ASD should evaluate whether physical therapy or spinal surgery can improve sitting posture and QOL, especially for those with high SVA or PT.

## Introduction

Adult spinal deformity (ASD) is a major socioeconomic problem in the aging population [1]. It is strongly associated with functional disability and reduced health-related quality of life (HRQOL) [2,3]. According to the International Spine Study Group (ISSG) [4,5], ASD is defined as the presence of at least one of the following standing radiographic criteria: Cobb angle ≥ 20°; sagittal vertical axis (SVA) ≥ 50 mm; pelvic tilt (PT) ≥ 25°, and thoracic kyphosis (TK) T1-T12 ≥ 60°. Traditionally, preoperative and postoperative spinal alignments have been assessed using standing radiographs [6,7].

Patients with ASD spend more time sitting than healthy individuals [8]. Therefore, understanding spinal alterations in this functional position is essential in daily clinical practice. It is well-known that the spine shapes differ significantly between sitting and standing postures [9]. Recent studies have focused on the importance of including ASD sitting radiographs in preoperative planning to better choose the upper instrumented vertebra and avoid mechanical complications [8,10]. Despite the importance of assessing the sitting position in daily functionality and surgical treatment planning, to our knowledge, a thorough analysis of the sitting posture in patients with ASD has yet to be conducted.

This study aimed to describe spinopelvic adaptations in standing and sitting positions in patients with ASD who had different types of spinal deformities and to determine their relationship with HRQOL outcomes.

## Materials and methods

Participants

This study was cross-sectional and approved by the Institutional Review Board of our university (Saint Joseph University, Beirut, Lebanon, CEHDF1259). Patients with ASD, more than 20 years of age and presenting at least one of the following radiographic criteria were recruited: SVA >50 mm, PT >25°, pelvic incidence (PI) mismatch to L1-S1 lumbar lordosis (LL) PI-LL >10°, T1-T12 TK >60°, and/or Cobb angle >20° [4,5]. Individuals with a history of musculoskeletal surgery or neurological, rheumatic, infectious, neoplastic, or other motion disorders were excluded from the study.

Asymptomatic individuals (control group) were recruited from the staff members of our institution. They did not meet the above-mentioned radiographic criteria and had no pain or history of spinal or lower limb surgery. All participants were informed of the procedures and signed a written consent form before participation.

Patients with ASD were divided into three groups: ASD-front (patients with only frontal scoliosis at a Cobb angle >20°), ASD-hyper thoracic kyphosis (TK) (patients with only thoracic hyperkyphosis TK >60°), and ASD-sag (patients with sagittal deformities, PT >25° and/or SVA >50 mm and/or PI-LL mismatch >10°).

Data collection

**Figure 1:**
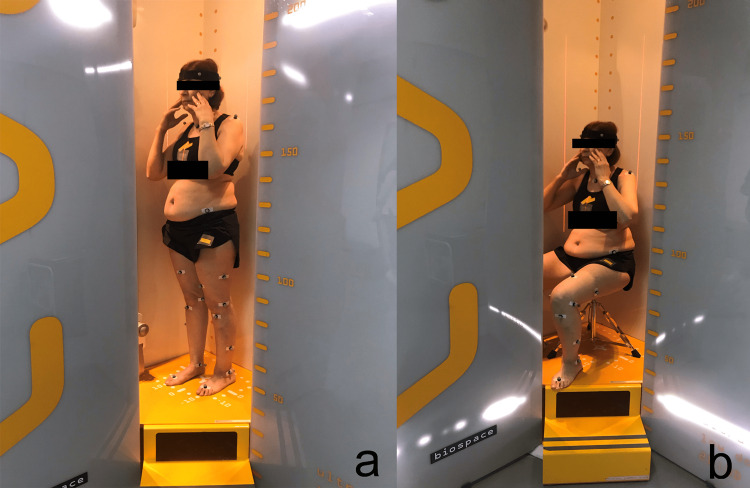
Low-dose biplanar radiograph acquisitions: standing (a) and sitting (b) positions. Authors’ own creations

Demographic data were collected, including age (years), sex (M/F), height (cm), and weight (kg). All participants underwent biplanar low-dose whole-body radiographs in the freestanding position (EOS Imaging®, Paris, France) [11,12]. Participants were asked to stand comfortably before moving their hands on the cheeks while maintaining their head and trunk in the same position [13,14]. Participants adopted the same hand position for sitting radiographic acquisitions on a height-adjustable backless stool, with hips and knees flexed at 90° (Figure 1).

**Figure 2:**
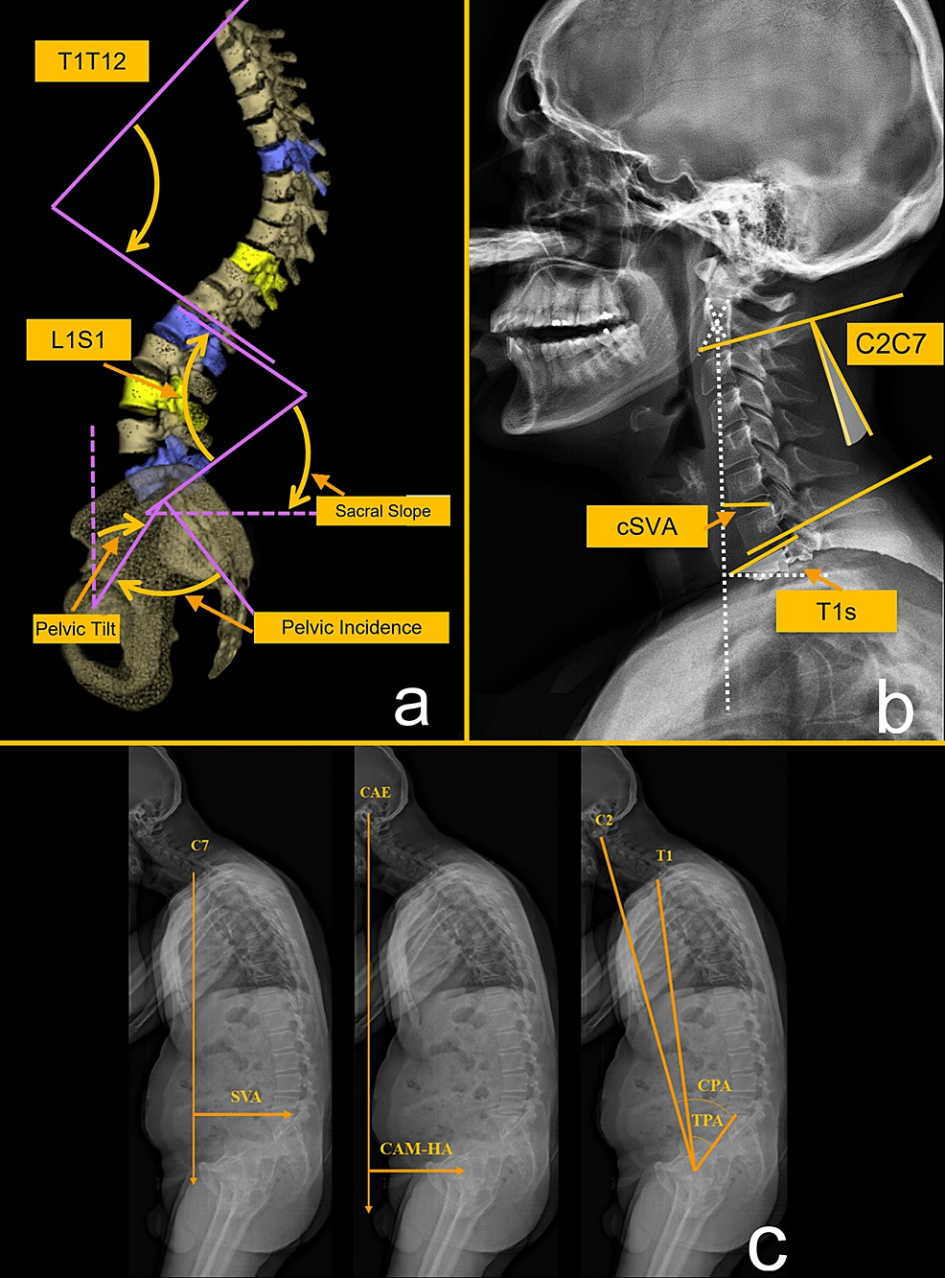
Radiographic parameters: spinopelvic (a), cervical (b), and postural (c). Authors’ own creations T1-T12: TK, L1S1: LL, cSVA: cervical sagittal vertical axis, C2C7: cervical lordosis, T1s: T1 slope, SVA: sagittal vertical axis, CAM-HA: center of the auditory meatus-hip axis, CPA: cervical pelvic angle, TPA: thoracic pelvic angle.

Three-dimensional reconstructions of the spine and pelvis were performed by trained operators using the SterEOS® software (version 1.6.5.8188, EOS Imaging®, Paris, France). The following spinopelvic parameters were calculated in both the standing and sitting positions: pelvic tilt (PT, the angle created by a line running from the sacral endplate midpoint to the center of the bifemoral heads and the vertical axis), pelvic incidence (PI, the angle between a line perpendicular to the sacral plate at its midpoint and a line connecting the same point to the center of the bicoxofemoral axis), sacral slope (SS, the angle between the superior plate of S1 and a horizontal line), L1-S1 lumbar lordosis (LL), PI-LL mismatch, T1-T12 TK, C2-C7 cervical lordosis (CL), T1 slope (T1S), and cervical SVA (cSVA). In addition, the following postural parameters were collected: SVA, the center of auditory meatus to hip axis (CAM-HA), thoracic pelvic angle (TPA), and the cervical pelvic angle (CPA) (Figure 2).

Spinopelvic flexibility was defined as the difference (Δ) between the radiographic parameters calculated in standing and sitting positions (Δ = standing − sitting).

All participants completed the following HRQOL questionnaires: short form 36 (SF36) with its physical (PCS) and mental (MCS) components [15], Oswestry Disability Index (ODI) [16], Beck’s depression inventory (BDI) [17], and a visual analog scale (VAS) for pain intensity.

Statistical analysis

Demographic data, standing and sitting radiographic spinopelvic parameters, flexibility, and HRQOL scores were compared between the four groups (ASD-sag, ASD-front, ASD-hyperTK, and controls) using the Kruskal-Wallis test, followed by Conover Iman for multiple pairwise comparisons. Correlations between the HRQOL scores and radiographic parameters were calculated using Pearson’s or Spearman’s coefficients. When necessary, the significance level was set at 0.05 and adjusted by a Bonferroni correction to accommodate for pairwise comparisons and multiple correlations. Statistical analyses were performed using Xlstat® (Addinsoft, Paris, France; version 2020.4.1).

## Results

Demographics and HRQOL scores

The ASD group included 95 participants, while 32 healthy adults formed the control group. Patients with ASD were older (52±19 vs 35±15 years, p<0.0001), with similar weight (71±14 vs 70±13 kg, p = 0.82) and a smaller height (161±10 vs 168±8 cm, p<0.001) than controls. The proportion of women with ASD was significantly higher than that of men (76% vs 40%, p = 0.003). The ASD group was subdivided into 40 ASD-sag, 24 ASD-front, and 31 ASD-hyperTKs.

The ASD-sag group exhibited the most significant changes (Table 1). Compared to controls, they had a decreased SF36-PCS score (35.7±9.3 vs 50.2±8.1, p<0.001). Their ODI scores indicated severe disability, whereas their BDI scores indicated mild depression. The VAS score indicated moderate pain. These alterations were found to a lesser degree in ASD-hyperTK and were less pronounced in the ASD-front.

**Table 1:** Comparison of HRQOL scores between the four subgroups. *: Significant difference. NS: not significant. SF36: short form 36, PCS: physical component summary, MCS: mental component summary, ODI: Oswestry Disability Index, VAS: visual analog scale, BDI: Beck’s depression inventory, ASD: adult spinal deformity, hyperTK: hyper thoracic kyphosis, sag: sagittal.

	ASD-front	ASD-hyperTK	ASD-sag	Controls	p	Controls	Controls	Controls	ASD-front	ASD-front	ASD-hyperTK
						vs	vs	vs	vs	vs	vs
						ASD-front	ASD-hyperTK	ASD-sag	ASD-hyperTK	ASD-sag	ASD-sag
SF36-PCS	45.5 ± 9.6	39.6 ± 7.5	35.8 ± 8.7	50.4 ± 7.7	<0.001	NS	*	*	NS	*	NS
SF36-MCS	47.8 ± 6.8	49.7 ± 9.8	51.6 ± 8.8	53.6 ± 8.5	0.06	NS	NS	NS	NS	NS	NS
ODI	20.7 ± 20.1	29 ± 18.2	39.6 ± 17.8	2.8 ± 4.9	<0.001	*	*	*	NS	*	*
VAS	4 ± 2.4	6.1 ± 2.6	6.6 ± 2.4	1.2 ± 0.5	<0.001	*	*	*	*	*	NS
BDI	9.2 ± 6.5	11.8 ± 8.4	12.7 ± 10.5	2.6 ± 3.7	<0.001	*	*	*	NS	NS	NS

Comparison of spinopelvic parameters in the standing position

The pelvic incidence was comparable between the four groups. The ASD-sag group had a higher pelvic retroversion than the other groups (27.3±11.3° vs ASD-front: 11.4±7.6°, ASD-hyperTK: 13.2±6.6°, controls: 10.5±6°, p<0.001). The increased PT in the ASD-sag group was coupled with a reduced LL (34.9±23.4° vs ASD-front: 56.8±9.1°, controls: 59.6±8.9°, p<0.001). The ASD-sag group tended to have a more pronounced positive sagittal imbalance than the other groups with a higher SVA (78.2±62.8 mm vs ASD-front: −4.2±23.2 mm, ASD-hyperTK: −0.2±22.6 mm, controls: 1.8±18.1 mm, p<0.001, Tables 2, 3).

On the other hand, the ASD-hyperTK group presented accentuated thoracic and lumbar curvatures with a higher TK compared to the other groups (72.4±10.9° vs ASD-sag: 42.6±22.6°, ASD-front: 37.1±13.3°, controls: 39±8.4°, p<0.001) as well as a higher LL (69.4±9.7° vs ASD-front: 56.8±9.1°, controls: 59.6±8.9°, p<0.001).

The ASD-front group had a more significant frontal Cobb compared to the other groups (38.2±14.7° vs ASD-sag: 21.5±18.2°, ASD-hyperTK: 8.9±9.3°, controls: 7.3±6°, p<0.001).

At the cervical level, CL was increased in ASD-hyperTK compared to controls and ASD-front (ASD-hyperTK: 20.7±14.5°, controls: 6±10.6°, ASD-front: 3.4±10.9°, p<0.001). T1s were higher in the ASD-hyperTK and ASD-sag groups than in the ASD-front and control groups (p<0.001). The cSVA differed only between the ASD-sag and ASD-front.

**Table 2:** Comparison of the radiographic parameters between the four groups (part 1). *: Significant difference, NS: not significant. SVA: sagittal vertical axis, CAM HA: center of auditory meatus-hip axis, PI: pelvic incidence, SS: sacral slope, PT: pelvic tilt, LL: lumbar lordosis, TK: thoracic kyphosis, ASD: adult spinal deformity, hyperTK: hyper thoracic kyphosis, sag: sagittal.

		ASD-Front	ASD-hyperTK	ASD-sag	Controls	p	Controls	Controls	Controls	ASD-front	ASD-front	ASD-hyperTK
							vs	vs	vs	vs	vs	vs
							ASD-front	ASD-hyperTK	ASD-sag	ASD-hyperTK	ASD-sag	ASD-sag
SVA (mm)	Standing	−4.2 ± 23.2	−0.2 ± 22.6	78.2 ± 62.8	1.8 ± 18.1	<0.001	NS	NS	*	NS	*	*
	Sitting	37.6 ± 20.7	40.8 ± 33	62.2 ± 38.6	47.5 ± 26.2	0.03	NS	NS	NS	NS	*	NS
	Flexibility	−41.8 ± 26.5	−41 ± 29.8	16 ± 50.7	−45.8 ± 27.7	<0.001	NS	NS	*	NS	*	*
CAM-HA (mm)	Standing	−19.7 ± 25.9	−9.8 ± 26	48.5 ± 75.9	−11.3 ± 21.1	<0.001	NS	NS	*	NS	*	*
	Sitting	−16.1 ± 25.7	3.2 ± 29.8	7.5 ± 44.3	−5.6 ± 32.3	0.06	NS	NS	NS	NS	NS	NS
	Flexibility	−3.6 ± 33.4	−13 ± 32.7	40.9 ± 69.6	−5.7 ± 31.7	<0.001	NS	NS	*	NS	*	*
PI	Standing	49.2 ± 6.5	50.4 ± 11.3	53.4 ± 13.7	51.3 ± 9.2	0.37	NS	NS	NS	NS	NS	NS
	Sitting	51.9 ± 8.7	50.9 ± 12.6	56.4 ± 13	49.9 ± 11.7	0.162	NS	NS	NS	NS	NS	NS
	Flexibility	−2.7 ± 4.3	−0.5 ± 5.1	−3 ± 11.5	1.4 ± 11.4	0.085	NS	NS	NS	NS	NS	NS
SS	Standing	37.8 ± 6.7	37.1 ± 8.8	25.9 ± 13.8	40.8 ± 6.9	<0.001	NS	NS	*	NS	*	*
	Sitting	21.4 ± 8.7	22 ± 12.9	16.2 ± 16.9	15.3 ± 9.7	0.113	NS	NS	NS	NS	NS	NS
	Flexibility	16.3 ± 9.3	15.1 ± 11.1	9.7 ± 12.9	25.4 ± 10.8	<0.001	*	*	*	NS	NS	NS
PT	Standing	11.4 ± 7.6	13.2 ± 6.6	27.3 ± 11.3	10.5 ± 6	<0.001	NS	NS	*	NS	*	*
	Sitting	30.4 ± 9.8	28.9 ± 13.2	40.2 ± 14.2	34.4 ± 11	0.002	NS	NS	NS	NS	*	*
	Flexibility	−19 ± 9.7	−15.6 ± 11.1	−12.9 ± 12.5	−23.9 ± 11.5	0.001	NS	*	*	NS	NS	NS
Frontal Cobb	Standing	38.2 ± 14.7	8.9 ± 9.3	21.5 ± 18.2	7.3 ± 6	<0.001	*	NS	*	*	*	*
L1S1 (LL)	Standing	56.8 ± 9.1	69.4 ± 9.7	34.9 ± 23.4	59.6 ± 8.9	<0.001	NS	*	*	*	*	*
	Sitting	31.5 ± 11.3	46.6 ± 19.1	31.5 ± 18.8	22.4 ± 12.6	<0.001	NS	*	NS	*	NS	*
	Flexibility	25.3 ± 12.2	22.8 ± 14.3	3.4 ± 18.7	37.1 ± 14.3	<0.001	*	*	*	NS	*	*
T1T12 (TK)	Standing	41.9 ± 11.6	72.4 ± 10.9	45.8 ± 19.9	44.9 ± 8.2	<0.001	NS	*	NS	*	NS	*
	Sitting	36 ± 13.7	67.2 ± 11.9	47.4 ± 19	35.5 ± 8.5	<0.001	NS	*	*	*	*	*
	Flexibility	5.9 ± 5	5.2 ± 5.1	−1.7 ± 6.8	9.4 ± 5.3	<0.001	NS	*	*	NS	*	*

**Table 3:** Comparison of the radiographic parameters between the four groups (part 2). *: Significant difference, NS: not significant. PI: pelvic incidence, LL: lumbar lordosis, C2C7: C2C7 lordosis, T1S: T1 slope, cSVA: cervical sagittal cervical axis, CPA: cervical pelvic angle, TPA: thoracic pelvic angle, ASD: adult spinal deformity, hyperTK: hyper thoracic kyphosis, sag: sagittal.

		ASD-front	ASD-hyperTK	ASD-sag	Controls	p	Controls	Controls	Controls	ASD-front	ASD-front	ASD-hyperTK
							vs	vs	vs	vs	vs	vs
							ASD-front	ASD-hyperTK	ASD-sag	ASD-hyperTK	ASD-sag	ASD-sag
PI-LL	Standing	−7.6 ± 10.3	−19 ± 7.6	18.5 ± 19.1	−8.2 ± 9	<0.001	NS	*	*	*	*	*
	Sitting	20.4 ± 11.9	4.2 ± 18.2	24.9 ± 18.6	27.5 ± 12.8	<0.001	NS	*	NS	*	NS	*
	Flexibility	−28 ± 12.6	−23.3 ± 14.3	−6.4 ± 18.3	−35.8 ± 13.1	<0.001	NS	*	*	NS	*	*
C2C7	Standing	3.4 ± 10.9	20.7 ± 14.5	13.1 ± 18	6 ± 10.6	<0.001	NS	*	NS	*	*	NS
	Sitting	9.1 ± 12.8	22.1 ± 18.3	16.4 ± 16.5	7.3 ± 13.5	<0.001	NS	*	*	*	NS	NS
	Flexibility	−5.7 ± 12.4	−1.4 ± 15	−3.8 ± 16.7	−1.4 ± 8.5	0.721	NS	NS	NS	NS	NS	NS
T1S	Standing	25.4 ± 9.9	38.2 ± 10.5	36.1 ± 11.6	27 ± 8.2	<0.001	NS	*	*	*	*	NS
	Sitting	27. 3 ± 12	42.1 ± 10.7	35.7 ± 12.8	28.2 ± 9.6	<0.001	NS	*	*	*	*	NS
	Flexibility	−1.8 ± 6.7	−4 ± 7.4	−1.5 ± 17.1	−1.3 ± 5.9	0.111	NS	NS	NS	NS	NS	NS
cSVA	Standing	2.1 ± 0.7	2.4 ± 1.5	3.1 ± 1.4	2.2 ± 0.9	<0.001	NS	NS	NS	NS	*	NS
	Sitting	2.1 ± 1.1	2.6 ± 1.5	3.3 ± 1.3	2.4 ± 1	<0.001	NS	NS	*	NS	*	NS
	Flexibility	−0.1 ± 0.9	−0.2 ± 0.5	−0.3 ± 1.6	−0.1 ± 0.5	0.785	NS	NS	NS	NS	NS	NS
CPA	Standing	9.9 ± 7.1	6.9 ± 7.6	26.7 ± 16	9.2 ± 6.2	<0.001	NS	NS	*	NS	*	*
	Sitting	28.1 ± 12	16 ± 16	35.6 ± 18.6	33 ± 13.4	<0.001	NS	*	NS	NS	NS	*
	Flexibility	−18.2 ± 9.9	−9 ± 11.1	−9.4 ± 13.4	−23.8 ± 12.8	<0.001	NS	*	*	*	*	NS
TPA	Standing	7.7 ± 6.5	6.6 ± 5.4	23.7 ± 14.8	7 ± 5.9	<0.001	NS	NS	*	NS	*	*
	Sitting	25.6 ± 10.6	15.2 ± 13.1	32.4 ± 16	30.2 ± 12.3	<0.001	NS	*	NS	NS	NS	*
	Flexibility	−17.8 ± 9.7	−8.6 ± 11.1	−8.7 ± 12.2	−23.2 ± 12.4	<0.001	NS	*	*	*	*	NS

Comparison of spinopelvic parameters in the sitting position

In the sitting position, the ASD-sag group presented a higher pelvic retroversion compared to the three other groups (40.2±14.2° vs controls: 34.4±11°, ASD-front: 30.4±9.8°, ASD-hyperTK: 28.9±13.2°, p = 0.02). SVA was higher in ASD-sag compared to the ASD-front group (62.2±38.6 mm and 37.6±20.7 mm, p = 0.03, Tables 2, 3).

LL and TK differed significantly among the four groups, with the highest values ​​in the ASD-hyperTK group (46.6±19.1° and 67.2±11.9°, respectively).

At the cervical level, the ASD-hyperTK group had higher cervical lordosis (22.1±18.3°) and T1s (42.1±10.7°) than the control group (7.3±13.5° and 28.2±9.6°, p<0.001). Patients with ASD-sag had a higher cSVA than controls.

Comparison of Spinopelvic Flexibility

Overall, patients with ASD-sag presented less pelvic mobility with a reduced ΔPT than the control group (−12.9±12.5° vs −23.9±11.5°, p<0.001; Tables 2, 3). Similarly, spinal curvatures showed fewer variations in the ASD-sag group: ΔLL and ΔTK were significantly reduced compared to the other groups (3.4±18.7° and −1.7±6.8°, respectively) vs controls (37.1±14.3° and 9.4±5.3°, respectively, p<0.001). In addition, patients with ASD-sag had a backward movement of the trunk and head with positive ΔSVA, unlike the other groups (16±50.7 mm vs controls: −45.8±27.7 mm, p<0.001). No significant differences were noted in the cervical parameters.

Patients with ASD-hyperTK presented lower flexibility in their pelvic retroversion (ΔPT: −15.6±11.1° vs −23.9±11.5° in controls, p<0.001) and LL (ΔLL: 22.8±14.3° vs 37.1±14.3° in controls, p<0.001).

Correlation with quality of life (QOL) scores

Significant correlations were found between the different HRQOL scores, the radiographic parameters, and the flexibility, with coefficients varying between 0.19 and 0.52 (Tables 4, 5).

**Table 4:** Correlations between radiographic parameters and HRQOL scores (part 1). NS: not significant. SVA: sagittal vertical axis, CAM HA: center of auditory meatus-hip axis, PI: pelvic incidence, PT: pelvic tilt, LL: lumbar lordosis, TK: thoracic kyphosis, SF36: short form 36, PCS: physical component summary, MCS: mental component summary, ODI: Oswestry Disability Index, VAS: visual analog scale, BDI: Beck’s depression inventory.

		SF36-PCS	VAS	ODI	BDI
						SVA	Standing	−0.45	0.34	0.52	0.17
							Sitting	−0.2	0.19	0.26	NS
Flexibility	−0.39	0.27	0.43	0.21		
CAM HA	Standing	−0.42	0.26	0.47	0.23
	Sitting	−0.3	0.23	0.23	NS
	Flexibility	−0.24	NS	0.34	0.22
PI	Standing	NS	NS	NS	NS
PT	Standing	−0.29	0.37	0.42	0.19
	Sitting	NS	NS	NS	NS
	Flexibility	−0.22	0.28	0.21	0.21
PI-LL	Standing	−0.23	0.23	0.35	NS
	Sitting	NS	NS	NS	−0.33
	Flexibility	−0.32	0.38	0.43	0.29
L1S1 (LL)	Standing	0.23	−0.21	−0.31	NS
	Sitting	NS	0.22	NS	0.39
	Flexibility	0.34	−0.4	−0.46	−0.29
T1T12 (TK)	Standing	−0.24	0.21	0.19	0.35
	Sitting	−0.35	0.32	0.32	0.4
	Flexibility	0.31	−0.32	−0.35	NS

**Table 5:** Correlations between radiographic parameters and HRQOL scores (part 2). NS: not significant. C2C7: C2C7 lordosis, T1S: T1 slope, cSVA: cervical sagittal cervical axis, CPA: cervical pelvic angle, TPA: thoracic pelvic angle, SF36: short form 36, PCS: physical component summary, MCS: mental component summary, ODI: Oswestry Disability Index, VAS: visual analog scale, BDI: Beck’s depression inventory.

		SF36-PCS	VAS	ODI	BDI
						C2C7	Standing	−0.28	0.21	0.25	NS
							Sitting	−0.27	NS	0.21	NS
Flexibility	NS	NS	NS	NS		
T1S	Standing	−0.34	0.25	0.34	0.21
	Sitting	−0.33	0.24	0.32	0.19
	Flexibility	NS	NS	NS	NS
cSVA	Standing	NS	NS	0.2	NS
	Sitting	−0.26	0.19	0.27	NS
	Flexibility	0.21	NS	NS	NS
CPA	Standing	−0.18	0.24	0.29	NS
	Sitting	NS	NS	NS	NS
	Flexibility	−0.28	0.32	0.28	NS
TPA	Standing	−0.22	0.28	0.34	NS
	Sitting	NS	NS	NS	NS
	Flexibility	−0.3	0.35	0.31	NS

**Figure 3:**
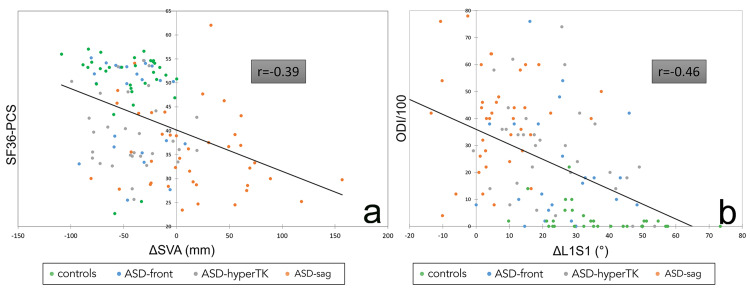
Examples of correlations between “flexibility” parameters and HRQOL scores: correlation between SVA flexibility ΔSVA and SF36-PCS (a); correlation between LL flexibility ΔLL and ODI (b). Authors’ own creations HRQOL: health-related quality of life, ASD: adult spinal deformity, hyperTK: hyper thoracic kyphosis, SVA: sagittal vertical axis, sag: sagittal, SF36: short form 36, PCS: physical component summary, LL: lumbar lordosis, ODI: Oswestry Disability Index.

The ODI score was positively correlated with the flexibility of the SVA (0.52). The physical component of the SF36 (PCS) correlated negatively with the flexibility of the SVA (−0.45, Figure 3).

## Discussion

This study evaluated spinopelvic adaptations in patients with ASD in standing and sitting postures. While clinical assessment in ASD is usually based on HRQOL scores and radiographic parameters evaluated in standing posture, the sitting position is a functional posture frequently adapted, especially by patients [18-20]. Our study showed that spinopelvic flexibility between standing and sitting differs according to the type of spinal deformity in ASD. Patients with sagittal malalignment were the most affected, demonstrating little flexibility with altered posture in the sitting position. These changes correlated with the participants’ HRQOL scores.

When standing, the ASD-sag group presented a classical sagittal imbalance [21-23]. A decrease in LL was observed with increased PI-LL mismatch. This decrease results in an anterior sagittal imbalance, leading to compensatory pelvic retroversion. Due to the inability of the compensation mechanisms to restore sagittal balance, altered global balance parameters were observed with increased SVA, CAM-HA, and CPA, indicating that the individuals in this group had their trunk and head shifted anteriorly. To maintain horizontal gaze, the T1 tilt increases, increasing cervical lordosis and cSVA.

In contrast, subjects with isolated hyperkyphosis (ASD-hyperTK) could compensate for their sagittal imbalance by increasing LL, leading to an SVA similar to controls without needing pelvic retroversion. This was sufficient to maintain the overall balance parameters within the normal limits. However, maintaining a horizontal gaze required increased cervical lordosis, similar to ASD-sag.

Patients with isolated scoliosis (ASD-front), not associated with a sagittal imbalance, showed no postural alteration compared to the control group.

Control individuals performed pelvic retroversion in the sitting position with flattening thoracic and lumbar curvatures. This positional hyperlordosis was more significant than the increase in pelvic retroversion (ΔLL = 37° vs ΔPT = −24°), resulting in a forward trunk shift and increased SVA, CAM-HA, and CPA compared to the standing position. However, these postural changes did not affect the horizontal gaze and cervical spine parameters.

**Figure 4:**
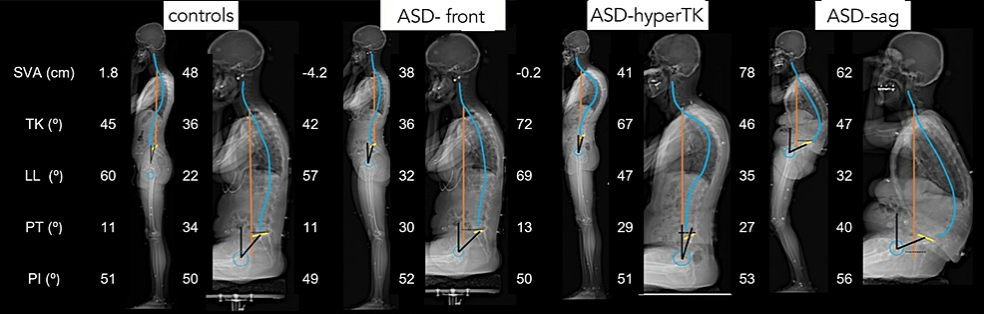
Sitting postural adaptations in the four groups: control, ASD-front, ASD-hyperTK, and ASD-sag. ASD: adult spinal deformity, hyperTK: hyper thoracic kyphosis, sag: sagittal.

In contrast, patients with sagittal imbalance (ASD-sag) showed limited pelvic flexibility toward retroversion (decreased ΔPT), probably related to excessive PT already encountered in the standing position. This limited pelvic flexibility might indicate that patients with sagittal imbalance have already used their retroversion reserve while standing. However, these patients had a higher PT while sitting than the other groups (Figure 4). Flattening of the thoracic and lumbar curvatures was also less evident in patients with ASD-sag. This limitation of spinal flexibility could be related to a degenerative effect [24,25], especially because the ASD-sag group was the oldest in our population. Because the degree of flattening of the LL was insufficient compared to the degree of the PT (ΔLL = 4° vs ΔPT = −12°), patients with ASD-sag projected their trunk and head backward in the seated position, resulting in a decreased SVA and CAM-HA compared to the standing position. The lack of curvature flattening also resulted in fewer variations in CPA and TPA. However, these spinal alterations affected the maintenance of the horizontal gaze and required a significant increase in cervical lordosis, T1 tilt, and cSVA compared to controls.

Patients with isolated ASD-hyperTK could flatten their curvatures more than the ASD-sag group. However, elevated TK and LL were maintained when sitting compared to the controls. The harmonious decrease in thoracic and lumbar curvatures can explain the results obtained for the SVA and CAM-HA groups, which were comparable to those of the controls. This group showed less pelvic retroversion (ΔPT) variation than the control group. This result may be necessary to maintain the high LL required to compensate for their fixed thoracic hyperkyphosis.

Patients with isolated frontal scoliosis (ASD-front) showed similar results to controls, except for a decrease in the flattening of the LL in the sitting position, which might be attributed to rigidity at this level [26].

**Figure 5:**
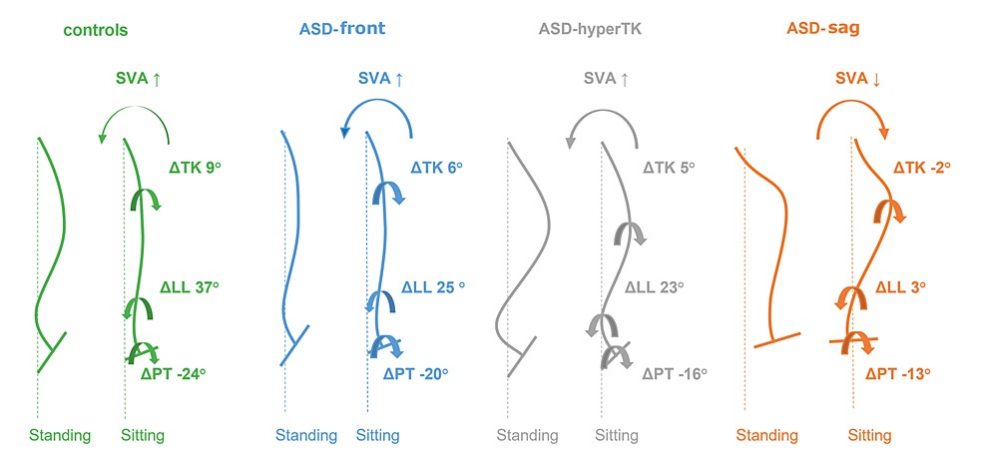
Schematic of seated position adaptations in the four groups: controls, ASD-front, ASD-hyperTK, and ASD-sag. ASD: adult spinal deformity, SVA: sagittal vertical axis, hyperTK: hyper thoracic kyphosis, sag: sagittal, LL: lumbar lordosis, PT: pelvic tilt.

Various adaptations of the seated position are presented in Figure 5. In summary, control individuals and patients with isolated scoliosis performed retroversion of the pelvis when seated. Given the flexibility of the spine, we noticed flattening of the curvatures, which was more pronounced in the lumbar region, tilting the trunk forward (increasing the SVA and CAM-HA). Similarly, subjects with isolated hyperkyphosis had a more flexible spine than those with ASD-sag, allowing them to achieve harmonious flattening, leading to the same result; they showed minor back flattening due to their fixed hyperkyphosis. In contrast, individuals with sagittal imbalance (ASD-sag) usually present with a rigid spine and cannot flatten their curves. They ended up with a backward trunk tilt (a decreased SVA) due to high pelvic retroversion.

Patients with spinal deformity have altered HRQOL scores [1,27,28]. We noticed that the ability to modify spinopelvic parameters when moving to a sitting position was correlated with better functionality scores (PCS and ODI) and less pain (VAS). Mental health scores (MCS and BDI) were less correlated with spinopelvic variations. This lessened correlation might reflect the lack of specificity of these scores, which are easily modified by other intrinsic and extrinsic factors [29].

The analysis of sitting radiographs in patients with ASD is not descriptive. The behavior of spinal curvatures in the sitting position is becoming essential for preoperative ASD planning [8,10,30]. Janjua et al. [10] found that relaxation of the unfused thoracic spine in the sitting position predicts a postoperative increase in kyphosis of the unfused thoracic segments. Yoshida et al. [8] highlighted the usefulness of preoperative sitting spinal alignment in patients with ASD. They found that the risk of mechanical complications, such as proximal junctional kyphosis, could be predicted using the distance from the planned upper instrumented vertebra to the C2 plumbline in preoperative sitting radiographs. Our study is the first to show how patients with ASD and different types of spinal deformities adapt their spinopelvic and postural alignment between the standing and sitting positions. These findings are essential for better understanding these biomechanical adaptations while planning surgery.

The major limitation of this study was the difference in demographic characteristics. To overcome this limitation, between-group comparisons were conducted while controlling for sex, age, and height using an analysis of covariance (ANCOVA) model. The same results were obtained for all comparisons.

## Conclusions

This study showed that patients with ASD exhibit alterations in spinopelvic adaptations between standing and sitting postures. Although all groups achieved pelvic retroversion while sitting, subjects with sagittal imbalance could not flatten their backs due to reduced lumbar and thoracic spine flexibility. Furthermore, a lack of flexibility seems to affect the comfort and physical abilities of patients with ASD. Our study is the first to describe spinopelvic and postural adaptations between standing and sitting positions in patients with ASD and different spinal deformities compared with controls. These findings should be considered in surgical planning and spinal fusion. Future studies on ASD should evaluate whether physical therapy or spinal surgery can improve sitting posture and QOL, especially for those with high SVA or PT.
